# Natural language processing in low back pain and spine diseases: A systematic review

**DOI:** 10.3389/fsurg.2022.957085

**Published:** 2022-07-14

**Authors:** Luca Bacco, Fabrizio Russo, Luca Ambrosio, Federico D’Antoni, Luca Vollero, Gianluca Vadalà, Felice Dell’Orletta, Mario Merone, Rocco Papalia, Vincenzo Denaro

**Affiliations:** ^1^ Department of Engineering, Unit of Computer Systems and Bioinformatics, Campus Bio-Medico University of Rome, Rome, Italy; ^2^ ItaliaNLP Lab, National Research Council, Istituto di Linguistica Computazionale “Antonio Zampolli”, Pisa, Italy; ^3^ R&D Lab, Webmonks S.r.l., Rome, Italy; ^4^ Department of Orthopaedic and Trauma Surgery, Campus Bio-Medico University Hospital Foundation, Rome, Italy; ^5^ Research Unit of Orthopaedic and Trauma Surgery, Campus Bio-Medico University of Rome, Rome, Italy

**Keywords:** natural language processing, deep learning, low back pain, spine disorders, artificial intelligence, systematic review

## Abstract

Natural Language Processing (NLP) is a discipline at the intersection between Computer Science (CS), Artificial Intelligence (AI), and Linguistics that leverages unstructured human-interpretable (natural) language text. In recent years, it gained momentum also in health-related applications and research. Although preliminary, studies concerning Low Back Pain (LBP) and other related spine disorders with relevant applications of NLP methodologies have been reported in the literature over the last few years. It motivated us to systematically review the literature comprised of two major public databases, PubMed and Scopus. To do so, we first formulated our research question following the PICO guidelines. Then, we followed a PRISMA-like protocol by performing a search query including terminologies of both technical (e.g., *natural language* and *computational linguistics*) and clinical (e.g., *lumbar* and *spine surgery*) domains. We collected 221 non-duplicated studies, 16 of which were eligible for our analysis. In this work, we present these studies divided into sub-categories, from both tasks and exploited models’ points of view. Furthermore, we report a detailed description of techniques used to extract and process textual features and the several evaluation metrics used to assess the performance of the NLP models. However, what is clear from our analysis is that additional studies on larger datasets are needed to better define the role of NLP in the care of patients with spinal disorders.

## 1. Introduction

Low Back Pain (LBP) is a particular condition of “pain and discomfort localized below the costal margin and above the inferior gluteal folds, with or without leg pain” as defined in the European Guidelines for Prevention of Low Back Pain [1]. Based on the onset, such condition may be either classified as acute or chronic. Events of the former category usually occur suddenly, lasting no more than six weeks, often associated with trauma. We refer to chronic LBP if the pain lasts more than twelve weeks, caused by a large pool of diseases like disc degeneration and herniation, spondyloarthritis and spondylolisthesis. In many cases, chronic LBP is treated with spine surgery, involving several risks for the patient, including persisting pain, incidental dural tears, vascular injuries, and infections.

The prevalence of such a musculoskeletal condition is increasing world-wide. A recent study [2] has reported the number of people experiencing LBP at some point in their lives increased from 377.5 million in 1990 to 577.0 million in 2017, globally. Even if the prevalence increases with age, a large amount of people experiences LBP not only in their earlier adulthood but also during adolescence [3]. In particular, chronic LBP is often considered the main reason for disability in a large portion of the population [4]. Even in cases in which pain does not imply disability, this condition often causes activity limitation and work absence [5,6], leading to a high economic burden on workers, industries, and governments [7]. All these aspects concerning LBP and, more in general, related spine disorders, pose a particular attention towards the care of this condition.

In recent years, the most ground-breaking technologies have been explored in the care of LBP, including Artificial Intelligence (AI) and Computer Science (CS), which have seen their application in the care of LBP in several studies [8,9]. A promising trend in this field involves Natural Language Processing (NLP), a discipline at the intersection between CS, AI, and Linguistics. NLP leverages unstructured texts written in the human-interpretable (natural) language. In recent years, NLP has already been applied in health-related domains, from radiology [10] to oncology [11], ranging from health-specific tasks, such as classifying medical notes from the clinical notes [12], to more traditional ones, such as opinion mining on patients’ reviews [13]. Recently, another review has focused on NLP in chronic diseases [14] in which, differently from our work, the authors did not focus on any spine disorder. However, the combination of NLP and healthcare is progressively gaining momentum, and has also been investigated in LBP care models. In this study, we have systematically reviewed the available literature on the application of NLP to develop innovative tools for diagnosing and treating LBP. Our aim is to describe the state of the art of such technology and identify future directions and potential implementations.

## 2. Materials and Methods

To perform an exhaustive overview of the applications of NLP in the management of LBP we interrogated both PubMed and Scopus databases with similar queries. For both databases, we performed the search on November 6th, 2021.

### 2.1. Research question

AI and CS systems have already been shown to be a great support to physicians in the task of diagnosing and treating LBP and related pathologies in humans [8,9]. With this work, we aimed to provide a comprehensive review of the literature regarding the described applications of NLP-related methods to the care of patients affected by LBP. Precisely, following the *PICO* guidelines, we aimed to answer the following research question:

- In human subjects, no matter for any demographic information, affected by LBP and related spine disorders {**P**opulation/**P**roblem}
- may NLP-related methodologies, {**I**ntervention}
- compared with human operators and already existing tools, {**C**omparison}
- help healthcare providers in the management of such conditions? {**O**utcome}

### 2.2. Research protocol

To perform an exhausting review of the literature, we developed the following research protocol. First of all, we elaborated a search query. Then, we formalized the inclusion/exclusion criteria. We performed the query on two public databases, namely PubMed and Scopus. In both databases, we performed the query on the title and the abstract of the articles. For the Scopus database, in addition, we also considered the keywords assigned to the papers. After conducting the first screening by removing the duplicated articles, two authors carried a preliminary screening after reviewing titles and abstracts (and, eventually, the keywords) of the total amount of papers. After that, the same authors went deeper by analyzing full-text articles. During the previous steps, we excluded papers not meeting the inclusion criteria from further analyses. Whenever a discordance happened, the two authors discussed it together until reaching a consensus. Finally, we reported in the present review the works retrieved.

**Figure 1:**
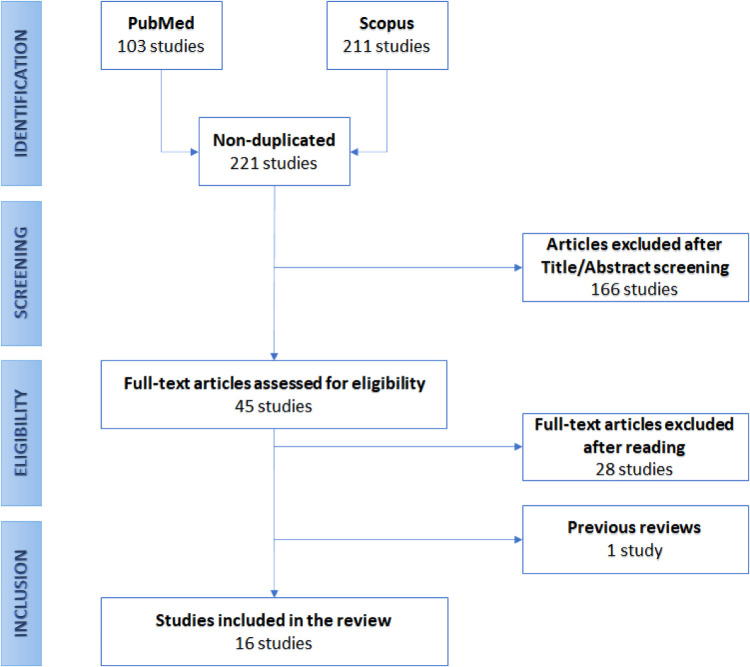
Preferred reporting items for systematic reviews and meta-analyses (PRISMA) flow diagram.

The developed protocol is resumed in Figure 1, reporting the flow-chart diagram realized according to the *PRISMA* protocol.

### 2.3. Search query

The proposed search query was divided into two different parts, one including terms from the NLP terminology, the other including terms related to LBP. In each of the two query sections, the terms have been linked by the logical OR operation, while the inter-relation between the two parts has been represented by the logical AND operation, meaning that the papers resulting from the interrogation had to present at least one of the terms for both query sections.

The NLP part contained several terms, each belonging to a particular characteristic of the NLP methodologies. Of course, terms as *natural language*, *NLP*, *NLG* (an acronym for NL Generation), and *NLU* (standing for NL Understanding) were directly inherent to the scope. Terms like *computational linguistics* and *text mining* were included because directly related to the NLP field, and often utilized as interchangeable synonyms. For both of them, there are only slight differences. Sometimes, field practitioners disagree about those differentiations. Usually, computational linguistics concerns the development of computational models to study some linguistic phenomenon, also concerning other fields such as sociology, psychology, and neurology. For example, a successful CL approach may be designing a better linguistic theory of how two languages are historically related. NLP, instead, is mainly oriented towards solving engineering problems analyzing or generating natural language text. Here, the success of the NLP approach is quantified on how well the developed system resolves the specific task. Text mining, instead, usually refers to turning unstructured text into structured data to further exploit it, e.g. through statistical analysis (data mining). Some practitioners find NLP is a part of text mining. However, there is still not a consensus about it.

Instead, terms as *tokenization*, *word embedding*, *rule based*, *regex*, *regular expression*, *bert*, and *transformers* refer to the methods to pre-process, extract features and models used to elaborate unstructured text, while *automated reporting*, *summarization*, *named entity recognition*, and *topic model* refer to specific tasks that can be performed on the text and are typical in the medical domain. Furthermore, we included some other generic terms: *text analysis*, *free text*, *biomedical text*, *medical text*, *clinical text*, *biomedical notes*, *medical notes*, *clinical notes*; and *linguistics*.

The medical part, instead, contains all terms related to the LBP and spine disorders conditions: *low back pain*, *lumbar*, *intervertebral disc degeneration*, *intervertebral disc displacement*, *spondylarthritis*, *spondylolisthesis*, *disc herniation*, *spine surgery*, *spondylarthrosis*, and *durotomy*.

### 2.4. Inclusion and exclusion criteria

This systematic review aimed to gather all the studies concerning the utilization of NLP in the diagnosis, prevention, and treatment of LBP. Straightforwardly, all the selected articles had to meet all the following inclusion criteria:

- LBP must have been between the main topics of the articles;
- NLP techniques must have been used in the studies;
- Subjects of the studies: all the articles must have been based on studies of the human spine pathology;
- Language: all articles must have been written in English.

Conversely, we excluded articles that did not meet the inclusion criteria for one of the following reasons:

- Low Back Pain or spine diseases were not considered;
- No automatic tool of text analysis were exploited;
- Animal studies.

### 2.5. Quality of evidence

**Figure 2:**
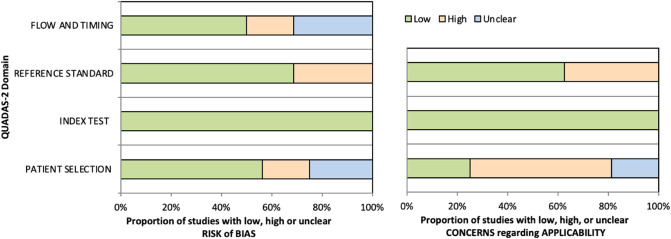
Summary of the methodological quality of included studies regarding the 4 domains assessing the risk of bias (left) and the 3 domains assessing applicability concerns (right) of the QUADAS-2 score. The portion of studies with a low risk of bias is highlighted in green, the portion with an unclear risk of bias is depicted in blue, and the portion with a high risk of bias is represented in orange.

The methodological quality of included studies was assessed independently by two reviewers (L.A. and F.R.), and any disagreement was solved by the intervention of a third reviewer (G.V.). The risk of bias and applicability of included studies were evaluated by using customized assessment criteria based on the Quality Assessment of Diagnostic Accuracy Studies (QUADAS-2) [15]. This tool is based on 4 domains: patient selection, index test, reference standard, and flow and timing. Each domain is evaluated in terms of risk of bias, and the first 3 domains are also assessed in terms of concerns regarding applicability. Sixteen studies were rated on a 3-point scale, reflecting concerns about risk of bias and applicability as low, unclear or high, as shown in Figure 2 (the details of the analysis are presented in Supplementary Tables S1 and S2).

## 3. Results

The searching queries were performed on November 6th, 2021, on two databases, namely PubMed and Scopus, resulting in 103 and 211 papers, respectively. Nonetheless, many of these articles were duplicates. So, as a first screening, we removed the repeated studies, resulting in 221 papers. Then, we analyzed the remaining articles’ titles and abstracts. In this phase, we excluded the works not meeting the inclusion criteria. This operation reduced the number of eligible articles to 45. Among them, we encountered one narrative review [16], in which Groot et al. recently focused on the role the NLP in spine surgery in six studies from the recent literature. However, since these papers are extensively reported in this review, we did not further focus on their work here. So, the final screening was performed by reading the full text of each paper, leading to retaining 16 of them. Figure 1 graphically shows the described selection process through a flow-chart diagram according to the PRISMA protocol.

In the following paragraphs, we analyze included studies particularly focusing on the tasks and models in which NLP is involved, also reporting the metrics used to evaluate the linguistic approaches.

### 3.1. Tasks

We identified three main NLP methodologies, namely classification, annotation, and prediction. Both first two approaches concern the identification of a category (class) to which a document belongs, differing for what the NLP methods are applied. In the classification approach, the system associates a label to each testing example (i.e., the patients’ document). A classification system may provide information about a diagnosis, as a Computer-Aided Diagnosis (CAD) system, which the physicians may exploit to decide, for example, whether or not to operate on a patient. Also, healthcare providers may utilize such a system to improve quality control, while researchers may use it to retrieve a large cohort of patients suffering from a particular condition and then conduct some research analysis.

In the annotation approach, NLP is used to label the documents, too. However, it is implemented as a part of the entire system, thought to provide the classification outcome from another kind of data, such as radiological images. From this point of view, the NLP system is a way to automatize the annotation of a large amount of data by identifying specific phenotypes related to a disease condition. In this way, the second part of the entire system may be trained and evaluated on a significant larger amount of data than the cases where only human annotations are considered. This kind of approach is used to develop successful predictors of clinical outcomes from clinical data and better define indications for surgery. It may improve clinical outcomes, which also leads to avoid invasive spine care and reduce costs.

**Figure 3:**
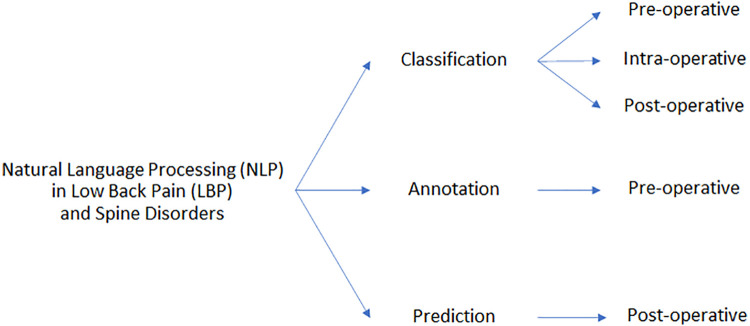
Schematic partitioning of the works concerning the application of NLP in LBP and related spinal disorders.

The third approach can be referenced as the identification of some category, too. However, here the scope is to predict some outcomes by exploiting previously acquired data (free-text notes, in this case). Healthcare providers may use such a system to predict some outcomes from the patients and thus arrange in advance the resources necessary for their care. Moreover, we further classified included studies based on the timeframe regarding surgical interventions. Thus, papers may also fall in the pre-, intra-, and post-operative task category, whether the task interests something before, during, or after surgery, respectively, as shown in Figure Figure 3.

#### 3.1.1. Classification

##### 3.1.1.1. Pre-operative tasks

We identified diverse studies in which the authors exploited pre-operative notes to identify useful diagnostic clues and findings. In detail, we retrieved:

- 1 paper focusing on the identification of multiple imaging findings;
- 1 paper focusing on the diagnosis of acute LBP;
- 2 papers focusing on the identification of spinal stenosis;
- 3 papers focusing on the identification of axial spondyloarthritis (axSpA);
- 1 paper focusing on the identification of type 1 Modic endplate changes.

Following, we describe the tasks.

*Imaging findings identification.* To advance the care of patients suffering from LBP, discovering distinct subgroups with similar prognoses and intervention recommendations is a relevant task. Spine imaging findings alone are often insufficient to diagnose the underlying causes of LBP. In addition, they are often not of clinical significance since their frequent occurrence in asymptomatic individuals [17]. To understand the relationships between imaging findings and LBP, an important step is the accurate extraction of the findings, such as spinal stenosis and disc herniation, from large patient cohorts. NLP may help identify lumbar spine imaging findings related to LBP in large sample sizes. Tan et al. [18] worked on this task.

*Acute LBP identification.* LBP events can be classified either as acute or chronic. While the former is usually treated with anti-inflammatories, with the recommendation of returning to perform daily activities soon, care of the latter often involves physical therapy, spinal injections [19] and even spine surgery. Thus, different conditions lead to different treatment recommendations, leading to different costs to the healthcare systems. Miotto et al. [20] faced this task.

*Identification of axSpA.* AxSpA is a serious spinal inflammatory disease characterized by the additional involvement of peripheral joints, entheses, and other systems (including the eye, the gut etc.) [21]. As patients with axSpA often present with peculiar imaging features, developing a tool to facilitate the identification of this subset of patients is a key step to achieve in improving the care of this condition. To exploit large datasets, NLP may be used to identify concepts related to axSpA in text, and thus create a cohort of patients with (high probability of having) the disease. Zhao et al. [22] and Walsh et al. [23] dealt with this this task. The last team also exploited their previous work in their [24] to identify axSpA patients.

*Stenosis identification.* Spinal stenosis is a condition of narrowing of the spaces within the spine, which can compress the spinal canal (spinal canal stenosis, SCS) and the nerve roots exiting at each intervertebral level (neural foraminal stenosis, NFS). Such conditions often develop in the lumbar spine. Here, NLP was used to classify both SCS and NFS, also with a severity grading scale [25,26].

*Type 1 Modic endplate changes identification.* Modic changes consist of magnetic resonance imaging (MRI) signal alterations affecting the endplates of the lumbar spine and are particularly frequent in patients with LBP [27]. For this reason, Huhdanpaa et al. [28] employed NLP to identify the Type 1 Modic changes from radiology reports.

##### 3.1.1.2. Intra-operative tasks

We identified diverse studies in which authors exploited operative notes to find evidence of some surgery complications. In detail, we retrieved two papers focusing on incidental durotomy (ID) identification and another one focusing on vascular injury (VI) identification. Such complications have potential implications for recovery, causing the length of stay and costs to increase. Thus, an automated system for surveillance of these events is relevant to healthcare providers.

*Incidental durotomy (ID) identification.* Incidental durotomy (ID) is a common intra-operative complication during spine surgery, occurring up to 14% of lumbar spine surgeries [29]. It is defined as an inadvertent tearing of the dura during surgery with cerebrospinal fluid (CSF) extravasation or bulging of the arachnoid [30]. The group of *Karhade and Ehresman* faced the problem of automatizing detection of ID events from operative notes [31,32].

*Vascular injury (VI) identification.* The terms vascular injury (VI) refers to the trauma of blood vessels (either an artery or a vein). It is a common event during spine surgery, often resulting in serious bleeding, thrombosis, and additional complications. Karhade et al. [33] dealt with the problem of detecting VI events from operative notes.

##### 3.1.1.3. Post-operative tasks

Classification in post-operative tasks serves to identify events occurring after the surgical intervention, such as venous thromboembolism (VTE). VTE results from the formation of a blood clot which may obstruct the blood flow locally (thus causing edema and pain) or may travel to distant sites causing local blood flow arrest (such as in pulmonary embolism). Dantes et al. [34] attempted to identify from post-operative radiology reports the occurrence of VTE in patients who underwent various kinds of surgeries, including spine surgery.

#### 3.1.2. Annotation

Among the included papers, two implemented NLP to annotate radiology images. Lewandrowski et al. [35] classified findings related to spinal stenosis (both SCS and NFS) from pre-operative reports, while Galbusera et al. [36] trained the NLP model to identify several spinal disorders. In both cases, the authors retrieved the annotations for radiology reports and then used them to label the related images. However, in the study by Galbusera et al., it was not possible to identify the timing with respect to surgery, since they included several types of disorders, as well as patients undergoing post-operative radiological examination and follow-up.

#### 3.1.3. Prediction

Prediction tasks focus on predicting post-operative outcomes. In their first paper, Karhade et al. [37], they attempted to identify required re-operations due to wound infections arising after lumbar discectomy, while in a subsequent study [38] they identified unplanned re-admissions of patients who underwent posterior lumbar fusion. Both the tasks were intended to refer to a period within 90 days.

### 3.2. Data

Data used in the analyzed studies is the free text from clinical notes. However, the kind of notes exploited by the authors may vary in dependence on the task the authors aimed to cover. A large proportion of papers used radiology reports. This is obvious for studies aiming at identifying imaging findings [18] and diagnose a specific condition [22,23,25,26,28,34], or at annotating images [35,36].

Other examples include operative notes, obviously used for the intra-operative tasks [31–34], and post-operative ones too [37,38]. Furthermore, the article from Karhade et al. [38], compared different kinds of clinical notes, including discharge summaries [22], and physicians and nursing notes. With the exception of [36], in which Galbusera et al. exploited notes in Italian, all other studies referred to notes written in English language.

### 3.3. Models

**Figure 4:**
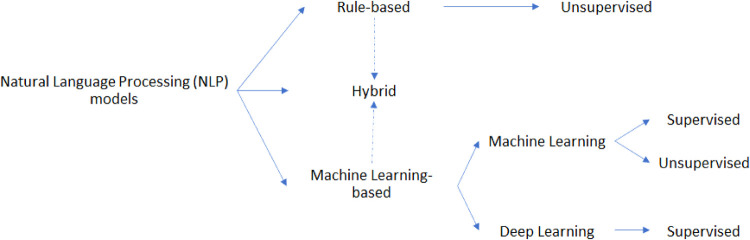
Schematic partitioning of the NLP models applied in LBP and related spine disorders.

The studies analyzed in this review used various kinds of NLP models. Referring to Figure 4, we identified such models as belonging to one of the following categories:

- *Rule-based* approach: exploits both linguistic and custom heuristic rules/patterns to make decisions on the input data
- *Machine Learning-based* approach: exploits statistical information from text to train the model to predict the right outcomes

Of course, some pipelines may exploit both the presented approaches, falling into the so-called *hybrid* approach category. Furthermore, the machine learning (ML)-based approach may be further split into two subcategories, grouping studies that used ML models and others which implemented deep learning (DL) paradigms.

Also, models may be categorized as belonging to:

- *Supervised* approach, which exploits labelled data to train the model;
- *Unsupervised* approach, in which the algorithm is not provided with any labelled data.

By taking into consideration the above definitions, it is reasonable to consider the rule-base models as belonging to the unsupervised class of algorithms, while the ML-based models may fall in both categories. Nonetheless, the supervised approach is usually more performant because the model learns directly from input-output pairs, while the unsupervised ones leverage only the input data. However, the former approach may require a lot of labeled data, a process that can be extremely time-consuming, requiring several human resources (annotators), especially for large datasets. Furthermore, in the healthcare field, annotators should necessarily have a degree of expertise in the domain. This is the same reason why NLP was used to automatize the annotation process of images in some of the analyzed studies.

#### 3.3.1. Rule-based models

Rule-based models are concerned about simple searches of keywords among the text of clinical notes, often by also developing regular expressions (regex). These rules may consist of both syntactic and semantic rules, also leveraging knowledge from both linguistics and the application domain (knowledge-driven approach). To identify (and then remove) negated occurrences, authors usually exploits algorithms such as NegEx [39]. This approach was implemented in [20] to identify acuity in LBP, and in [28] to identify Type 1 Modic changes, while in [18,25,26] to identify several findings related to LBP and stenosis from MRI and/or x-ray reports.

#### 3.3.2. Machine learning-based models

ML models are algorithms that leverage their experience on previously seen data to automatically improve their performance on some task. Thus, they leverage a data-driven approach, by learning discriminative content from a statistical representation of the input data. The authors of the paper encountered focused particularly on two models from the machine learning literature: Logistic Regression (LR) and eXtreme Gradient Boosting (XGBoost). The former was implemented in [20] for the acuity identification task and in [22] to identify axSpA. In both cases, the model was implemented together with a Least Absolute Shrinkage and Selection Operator (LASSO) regularization. The latter was particularly employed by Karhade et al. in several tasks [31–33,37,38]. Another used algorithm was the Support Vector Machine (SVM), employed in [23] to identify clues of axSpA and in [24] both to directly identify axSpA and to extract a feature for a multimodal random forest. Furthermore, authors in [34] exploited *IDEAL-X*, a tool introduced in [40] which exploits the online ML paradigm, to identify VTE following orthopedic surgery.

##### 3.3.2.1. Deep learning models.

The DL paradigm is a subfield of ML regarding the use of algorithms partly inspired by the brain structure and functioning, the so-called artificial (deep) neural networks. These algorithms are well known to perform better than ML in a large variety of applications. However, to be competitive they require a larger amount of training examples, and the training phase may be largely expensive in terms of time, especially when researchers do not have access to performant hardware facilities (i.e., Graphics Processing Units, aka GPUs). Probably for these reasons, only a few papers investigated the use of DL models. In [20], the authors compared a convolutional neural network (ConvNet) with classic ML and rule-based model. More recently, in [36] the authors fine-tuned a BERT [41] model pre-trained on general purpose italian text (“bert-base-italian-uncased”). Models like BERT are based on the Transformer’s architecture [42], introduced a few years ago. Exploiting a pre-trained Transformer-based model to initialize the weights and then train on some downstream tasks has become a standard practice within the NLP community.

##### 3.3.2.2. Unsupervised models.

All the above-reported studies leverage the supervised paradigm to train their models. The authors in [20] investigated the use of unsupervised models to identify acute LBP. They implemented a Latent Dirichlet Allocation (LDA) [43] to perform topic modeling, an unsupervised ML technique that captures patterns of word co-occurrences within documents to determine words’ sets clusters (i.e., the topics). They identified a set of keywords among the topics and then manually reviewed them to retain only those that seemed more likely to characterize acute LBP episodes. In other words, they selected the topics including most of the keywords with high probabilities. Then, they considered the maximum likelihood among these topics as the probability that a report referred to acute LBP. Furthermore, the authors in [22] exploited the so-called multimodal automated phenotyping (MAP) [44], to identify axSpA from related concepts and coded features.

#### 3.3.3. Hybrid models

For what concerns the hybrid paradigm, we encountered only one paper [18] exploiting it. Here, the authors implemented a logistic regression with elastic-net penalization leveraging several kinds of features. In particular, they also used features extracted with a combination of regex and NegEx.

### 3.4. Pre-processing

The pre-processing phase is dedicated to cleaning and elaborating input data. This step is necessary most of the time before feeding any algorithm in Computer Science and Artificial Intelligence approaches. Of course, NLP methods are not exempted.

Aside from tokenization (splitting the text into words, punctuation, etc.) and lower/upper-casing (normalizing words to their lower or upper-cased version), the most common procedures for text pre-processing are the following.

*Stop words removal.* Stop words are words highly common in a defined language, thus presenting the same likelihood to appear in both relevant and not relevant documents [45], i.e. carrying no informative content for the task in exam [28,31–33,37]. Also, some implemented the removal of generally less useful tokens, such as punctuation, numerals, and urls [20,37].

*Stemming.* Reduction of the words to their root form, usually by stripping each word of its derivational and inflectional suffixes [46]. Such a procedure aims to normalize the words from different inflections to a standard version [28,31–33,37].

*Lemmatization.* Similar to the stemming procedure, but instead of relying on heuristic chops of the words, leverage on vocabulary and morphological analysis of words to remove inflectional ending [20].

*Filtering.* This procedure discard words (or *n*-grams, i.e., a sequence of *n* words) occurring less than a fixed threshold in the entire (training) dataset. Because of their low prevalence, these words are not informative. This step reduces the number of misspelled words. The removal automatically reduces the dimensionality of word/document representations (i.e., the number of features), helping the model focus on the relevant features. Instead of discarding, the authors in [20] corrected to the terms in the vocabulary having the minimum edit distance (i.e., the minimum number of operations required to transform one string into the other).

### 3.5. Feature extraction

The term “feature extraction” refers to the procedure of combining variables from the data in order to provide a representation of each sample to be fed into (ML-based) models. The most common methods to extract features from text are:

*Bag of Words (BoW).* The Bag of Words model represents each document with a vector, in which every entry corresponds to the absence/presence (or the counting) of a specific word occurring inside that document [20,23,24,31,37]. The dimension of each vector is equal to the number of words encountered inside a corpus (e.g., a corpus built by the clinical notes collected). Given that, it is clear how the BoW representation is a sparse representation, i.e., every document shows a way greater number of absent words.

*Bag of *N*-grams (BoN).* The Bag of *N*-grams model is analogous to the BoW model. The only difference is that each feature is associated with an *n*-gram, i.e., a sequence of *n* words. Of course, several BoN models with different *n* may be combined together [20,23,24].

*Engineered features.* Features are extracted by leveraging the domain knowledge. For example, in [20] the authors retrieved a set of 5154 distinct n-grams based on concepts related to acute LBP episodes, while in [22] the number of occurrences of some concepts in free-text were used as features.

*Word embeddings.* Word embeddings are a way to encode the meaning of each word in a real-valued and non-spare vector representation. Models to retrieve this kind of representation, such as *word2vec* and *GloVe*, thus provide word representation such that the words with similar meaning or context are encoded in representations that are closer in the vector space. Thus, when a word has different meanings in the corpus, its representation is different depending on its context. This kind of feature extraction is exploited more whenever the final model consists of some neural networks, thus belonging to the DL-based approaches. In fact, we encountered word embedding features only in one paper [20] that explored the use of such an approach, using the *word2vec*’s *skip-gram* algorithm and a convolutional neural network. In [36] word embeddings are created internally by the BERT model and are initialized by the “bert-base-italian-uncased” pre-trained model.

### 3.6. Feature manipulation

With the term “feature manipulation” we indicate procedures adopted to regularize the features, thus improving their carried information (regularization strategy), or to reduce the feature space, in order to exploit in the next steps a reduced number of the most relevant features (feature selection). For the former case, the Term Frequency-Inverse Document Frequency (TF-IDF) strategy aims to assign to each term in a document *D* a weight that is directly proportional to the term frequency in *D* and is inversely proportional to the term frequency in all the documents of the corpus. In this way, it regularizes the features by balancing the rare ones with the most common ones. By the way, this method is applicable to both BoW and BoN models [20,31–33,37], and engineered features [20], too. For the latter case, it usually concerns discarding the features less representative in the (training) dataset. In the case of BoW and BoN features, this step is equal to performing a filtering step on the text during the pre-processing phase. However, in [23] the authors evaluated the discriminative power of each feature *w* in relation to each class *c* by the following equation

(1) $$D_{c}(w)=\frac{1-p(c)}{1-p_{w}(c)}$$

in which *p*(*c*) is the prevalence of class *c* among the training snippets and *p*_*w*_(*c*) is the prevalence of the class *c* among the training snippets containing the feature *w*. The features which occurred at least in two snippets and presents *D*_*c*_(*w*) ≥ 2 for every class *c* were retained.

### 3.7. Evaluation metrics

The metrics recognized in the analyzed papers can be divided into the following categories. The scope of this section is to help future research orientate them into a vast amount of metrics and choose the ones that better fit their research.

#### 3.7.1. Discrimination metrics.

This kind of metrics measures the model’s ability to map input data into separated classes. If the model employed is of the probabilistic kind (i.e., it outputs a probability instead of directly outputting the class), a threshold is applied to map the model’s output to the class labels. Several metrics fall into this category, each having a specific meaning. Following, we reported the most common ones encountered in our study. Since most classification tasks were binary, we report the binary version of such metrics for simplicity and brevity purposes. However, in most analyzed papers, multi-class problems (classifying a sample to one label out of several classes) were approached as more binary tasks.

The entire set of the found discrimination metrics can be achieved from the confusion matrix (Table 1), a table layout that correlates the actual conditions of the samples, positive (P) and negative (N), with the conditions predicted by the model (PP and PN). It allows to easily visualize the number of correct predictions, both true positives (TP) and true negatives (TN), and the number of ill-classified samples, both false positives (FP) and false negatives (FN).

**Table 1:** Confusion matrix.

		Predicted condition	
		PP	PN
Actual condition	P	TP	FN
	N	FP	TN

The first metrics we introduce are the True Positive and the True Negative Rates (TPR and TNR, respectively), as defined in Eq. 2. These measures quantify the ability of the model to classify the positive (and the negative) samples in the evaluation dataset. In the analyzed works, they are often indicated with other names. Usually, the name by which they are addressed depends on the field of application. In medicine works, it is not strange to find TPR and TNR reported as *sensitivity* and *specificity*, respectively, while, especially in AI-related papers, TPR is often presented as *Recall*.


(2) $$TPR=\frac{TP}{P}=\frac{TP}{TP+FN}\in [0;1]\;TNR=\frac{TN}{N}=\frac{TN}{TN+FP}\in [0;1]$$

Other useful metrics are the Positive and Negative Predict Value (PPV and NPV, respectively), as defined in Eq. 3. They quantify the ability of the model to not misclassify the negative (and the positive) samples in the evaluation dataset. Thus, the PPV metric is often referred to as *Precision*.


(3) $$PPV=\frac{TP}{PP}=\frac{TP}{TP+FP}\in [0;1]\;NPV=\frac{TN}{PN}=\frac{TN}{TN+FN}\in [0;1]$$

A more general metric, quantifying the general ability of the model to correctly classify the samples, independently by their actual condition, is the *Accuracy*, defined as in Eq. 4.


(4) $$Accuracy=\frac{TP+TN}{P+N}=\frac{TP+TN}{TP+FN+FP+TN}\in [0;1]$$

However, since this metric does not take into account a specific class, it is not very informative in case of a strong imbalance of the dataset. In fact, it is possible to show a high accuracy degree even when the model ill-classify all the samples belonging to the minority class. To clarify it, take into consideration the following example: we have 100 documents related to 100 patients; among these documents, only 3 samples belong to patients with an LBP diagnosis, while the others belong to the rest of healthy patients; if we classify each patient as healthy, we will still achieve an accuracy of 97%, which looks very good at a first impact, but it hides the fact that we are just predicting always the majority class. For this reason, it is good practice to prefer another metric but the accuracy, the **F*_1_-score*. The *F*_1_-score, also addressed as *F*_1_-measure, is the harmonic mean of precision and recall. It is defined as in Eq. 5, in which the score of the positive class is reported. The same score may also be computed for the negative class, by substituting Precision and Recall with their counterpart metrics, NPV and TNR.


(5) $$F_{1}=2\cdot \frac{TPR\cdot PPV}{TPR+PPV}\in [0;1]$$

Other widely used evaluation metrics are the Area Under the ROC and PCR Curves (AUROC and AUPCR, respectively), where ROC stands for Receiver Operating Characteristic and PCR stands for Precision-Recall Curve. Both curves are plotted considering the True Positive Rate against the False Positive Rate (*FPR* = *FP*/*P*), and the Positive Predict Value against the True Positive Rate, by considering the performances at different classification thresholds.

All of these metrics range between 0 and 1; the closer they are to the maximum value (i.e., 1), the more performant the system will be.

#### 3.7.2. Calibration metrics.

These kinds of metrics are a way to quantify the model’s ability to get close to the population underlying probability. While discrimination measures the predictor’s ability to separate patients with different responses, calibration captures the degree to which its numerical predictions match the outcomes [47,48]. In particular, some of the analyzed works reported intercept and slope measures [31–33,37,38] to assess the miscalibration of the system. Specifically, a positive/negative calibration intercept assesses the over-/under-estimation of the predictions, while a calibration slope evaluates the spread of the predictions; a slope greater/lower than 1 would indicate that the predictions are too moderate/extreme. For example, if *slope* < 1, the estimations are too high for patients who are at high risk and too low for patients who are at low risk [49]. However, calibration metrics are more relevant for clinical but computer science practitioners. Furthermore, the authors of the papers reporting calibration measures did not discuss their results in an exhaustive manner. Thus, in the analysis of these works (Section 4) we focused less on calibration metrics.

#### 3.7.3. Overall performance metrics.

They are a way to measure the overall performance of the probabilistic predictions, being correlated to both discrimination and calibration at the same time. In most of the papers, the overall performances were assessed through the *Brier Score*. Designed to assess the quality of the probability predictions in forecasting tasks [50], the score introduced by Brier can be exploited in tasks in which a model assigns probabilities to a set of *mutually exclusive* and *discrete* classes. Such a score is defined as follows:

(6) $$BS=\frac{1}{N}\sum_{i=1}^{N}\sum_{\;j=1}^{C}(\;p_{i,j}-y_{i,j}{)}^{2}\in [0;1]$$

In which we refer to *N* as the total number of samples for which the model is evaluated, to *C* as the total number of discrete classes, and *p*_*i*,*j*_ and *y*_*i*,*j*_ as the probabilistic outcome of the model and the actual class of the *j*th sample regarding the *i*th class, respectively. In particular, when the task is binary (*C* = 2), the Brier Score is equivalent to the *Mean Squared Error*:

(7) $$BS_{C=2}=MSE=\frac{1}{N}\sum_{i=1}^{N}(\;p_{i}-y_{i}{)}^{2}\in [0;1]$$

The Brier score may assume any value ranging between 0 and 1. However, being a measure of the prediction error, the closer it gets to the minimum value of the interval (i.e., 0), the more performant the model will be.

Another metric used to assess the overall performance is the *Standardized Net Benefit*. This decision curve analysis evaluates the clinical benefit of a predictive model over some default strategies across a range of threshold probabilities, defined as the minimum probability at which a patient/report is classified as presenting a particular condition [51]. In the analyzed papers reporting this decision curve analysis [31–33,37,38], classifying all the patients/reports as presenting the condition has been chosen as the default strategy. Also, comparisons with clinical gold standard codifications were present (see next paragraph).

#### 3.7.4. Comparative strategies.

When evaluating a predictive model, it is often important to have a comparison with the performance of other models. In some cases, the comparison is made with some baseline methodology considered as the gold standard in actual clinical practice, like Current Procedural Terminology (CPT) and International Classification of Diseases (ICD). For example, in [31] the authors compare their model with both the kind of codes for durotomy (i.e., CPT=63,707, 63,709, 63,710; ICD-9=349.3; ICD-10=G96.11, G97.4). To address it, all the previously described metrics can be used to compare two or more models. Also, a particular version of the Brier Score is the so-called *null-model Brier Score*. It is a version of the Brier Score computed on a virtual (baseline) model generating a predicted probability equal to the population prevalence of the outcome (=*P*/(*P* + *N*)). Another strategy to compare the two models is by evaluating the *p*-values after performing some statistical test, like McNemar’s one.

### 3.8. Explainability

AI is gaining momentum for a large number of different aspects of our society, including healthcare and will surely continue to have a significant influence in our daily lives the near future. However, current methods may achieve high performance of a specific task but often lack interpretability. The absence of more interpretable feedback together with the output from the model is a great inconvenience, especially in the clinical field. For what concerns the explainability, only *Karhade* and colleagues have addressed it, at both global and local (for the single subject) levels among included studies. It was possible thanks to the implementation of the XGBoost. Such an algorithm can provide the importance of each feature in a particular task. For example, in [31] the patient-level explanations were provided by highlighting the most important features (the words), used by the algorithm to detect ID, inside the text. Global explanations were provided averaging the importance score of each feature across all patients (the documents), to demonstrate the generally most important factors used for detection. Analogous reasoning was applied in their other works [37].

### 3.9. Softwares

We encountered several softwares and tools employed in the analyzed papers. The most used programming languages used to implement the NLP methods were *Java*, *Python*, and *R*. In particular, Java was used to implement rule-based models [18,28], also incorporating Apache Lucene (v 6.1.0) Application Program Interface (API), while Python and R [18,24–26] were usually exploited for ML approaches and conduct the statistical analyses.

Furthermore, various tools were used to perform manual annotations, such as REDCap [52] platform^1^ [18,28] and Visual Tagging Tool^2^ [23]. Also, in [36] the authors implemented a user interface with Python by exploiting the Python binding version of the graphical user interface toolkit Qt (PyQt^3^).

### 3.10. Domain-specific knowledge

**Figure 5:**
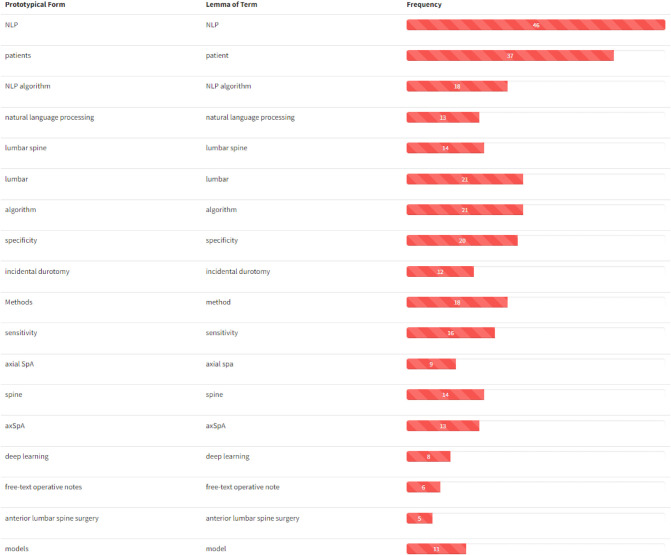
Glossary extracted from the abstracts of the papers included in this work. Entities are ranked following their domain relevance. For ease of visualization, only the first part of the glossary (containing the most relevant terms) is reported.

**Figure 6:**
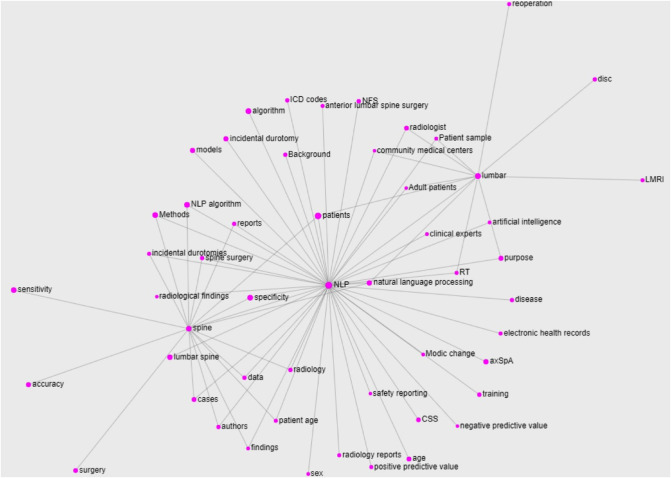
Knowledge graph built for the main entities of the domain extracted from the abstracts of the papers included in this work. For ease of visualization, only the terms with a frequency greater than 3 and the relations occurring at least twice are reported.

Perhaps unusual in works of this kind, we conducted a typical NLP analysis of the papers included in this review to extract some domain-specific knowledge from the articles included in this review. In particular, we treated the collection of abstracts as a corpus from which we extracted domain-specific entities to build its glossary. We then retrieved the relations between them to create the knowledge graph of the domain we can call *Natural Language Processing in Low Back Pain and Spine Disorders*. To do so, we applied the *T*2*K*^2^ suite of tools [53] to obtain the glossary in Figure 5, reporting the prototypical form of the entity (the term form most frequently attested in the corpus), its lemmatized form (Section (d)), and its frequency of occurrence. It is worth noting that these domain-specific entities may consist of single nominal terms but also of complex nominal structures. For ease of visualization, only the first part of the glossary (containing the most relevant terms) is reported in figure: the ranking follows the domain relevance of the entities, computed on the basis of their *C–NC* value [54]. By looking at the obtained glossary, it is easy to notice that the entities *NLP* (and its variations) and *lumbar spine* are the most relevant ones together with *patients*. We then selected these words as the most representative of the domain (we excluded the term *patients* because too generic) to compute their relations with the other entities in the glossary. In particular, the relations are computed on the basis of the co-occurrence of the entity in the core sentence (the one in which appear the entity under consideration) and the ones immediately before and after. The knowledge graph obtained with such entities and relations is reported in Figure 6. For ease of visualization, we filtered out terms with a frequency lower than 3 and the relations not occurring at least twice.

As interpretable from the figure, the *NLP* entity represents the core of the graph (and thus, in some sense, of the domain). It is worth noting the presence of the several diseases related to the *NLP* part (*incidental durotomies*, *axSpa*, *modic changes*, etc.), suggesting the obvious importance of these terms for the domain, and of the terms related to the computational part (*algorithm*, *models*, *artificial intelligence* etc.) and the data sources (*radiology reports*, *electronic health records*, etc.). However, both the *lumbar* and *spine* entities show a few prerogative relations, such as with *disc* and with *surgery*, respectively, that are not shared with the *NLP* core. Also, apart from the entity *natural language processing* that is just a variant of the *NLP* one, the only relation shared by all the three main entities is the one with *patients*. Besides being a very generic term, this result suggests the focus the authors put on the patients of their works, which also reflects the findings of the glossary.

## 4. Analysis

Our systematic review on the application of NLP to lumbar spine disorders eventually included 16 studies, whose main characteristics are summarized in Table 2. For the studies [18,20] using more than a model, we reported only the one with the best performance.

**Table 2:** Overview table of analyzed papers.

Study	Year	NLP task	Task category	Domain	Source	Model
Caton et al. [25]	2021a	Class.	pre-op.	SCS/NFS	Lumbar MRI reports	rule-based
Caton et al. [26]	2021b	Class.	pre-op.	SCS/NFS	Lumbar MRI reports	rule-based
Miotto et al. [20]	2020	Class.	pre-op.	acute LBP	Clinical notes	DL (ConvNet)
Walsh et al. [23]	2017	Class.	pre-op.	axSpA	Electronic medical records	ML (SVM)
Walsh et al. [24]	2020	Class.	pre-op.	axSpA	Clinical chart database	ML (SVM)
Zhao et al. [22]	2019	Class.	pre-op.	axSpA	Electronic medical records	ML (SAFE+MAP)
Huhdanpaa et al. [28]	2018	Class.	pre-op.	Type 1 Modic Endplate Changes	Lumbar MRI reports	rule-based
Tan et al. [18]	2018	Class.	pre-op.	LBP-related imaging findings	Lumbar MRI reports and X-ray reports	hybrid
Lewandrowski et al. [35]	2020	Annot.	pre-op.	SCS/NFS	Lumbar MRI reports	Not specified
Galbusera et al. [36]	2021	Annot.	/	spinal disorders	Lumbar X-ray reports	DL (BERT)
Ehresman et al. [32]	2020	Class.	intra-op.	Incidental durotomy	Electronic health records	ML (XGBoost)
Karhade et al. [31]	2020a	Class.	intra-op.	Incidental durotomy	Operative notes	ML (XGBoost)
Karhade et al. [33]	2021a	Class.	intra-op.	Vascular injury	Operative notes	ML (XGBoost)
Dantes et al. [34]	2018	Class.	post-op.	Venous Thromboembolism	Electronic medical records	ML (IDEAL-X)
Karhade et al. [37]	2020b	Pred.	post-op.	Reoperation due to infection	Operative notes	ML (XGBoost)
Karhade et al. [38]	2021b	Pred.	post-op.	Unplanned readmission	Operative notes	ML (XGBoost)

From a chronological point of view, Walsh et al. [23] were the first ones, in 2017, to apply NLP to LBP and related disorders. They first explored the axSpA language to manually select three terms that are predictive of such condition, namely “sacroiliitis”, “spond(*)”, and “HLA–B27 positivity,” and their expanded term variations via regular expressions. Then, they extracted snippets of text from clinical notes and radiology reports, where a snippet is defined as a section of text containing a clinically meaningful concept surrounded by its context. Finally, they implemented a Support Vector Machine (SVM) algorithm for each concept to classify each snippet as intending the presence of axSpA or not. To do so, they extracted bigram features from the snippets and performed a discriminative power-based feature selection. They evaluated the system in a 10-fold cross-validation fashion, reporting metrics separately for each concept at the percentage of 95% (confidence interval) for accuracy (91.1%, 93.5%, 97.2%), PPV (91.1%, 93.5%, 97.2%), and NPV (91.1%, 93.5%, 97.2%). Also, they evaluated the system on an independent test set achieving comparable results. In total, the annotation for 900 “sacroiliitis”-related snippets, for 1500 “spond(*)”-related snippets, and for 1500 “HLA–B271”-related snippets were collected. The authors re-used the three developed models in [24]. In particular, the “spond(*)” related model was directly implemented in the classification of the axSpA identification task. Also, the output of the three models together was used in combination with other 46 coded features in a second experiment, what they called the Full algorithm. These other variables were extracted from structured data such as diagnosis codes for axSpA, laboratory data relevant to axSpA, medications, and comorbidities). In this second case, applied NLP models can be also viewed as feature extraction methods. They evaluated sensitivity, specificity, PPV, and NPV for both the Full algorithm (87.5%, 91.7%, 79.5%, and 95.2%, respectively) and Spond algorithm (95.0%, 78.0%, 61.3%, and 97.7%, respectively). Results were evaluated at 95% CI, determined through bootstrapping, with sampling with replacement of the observed data for 500 times. In total, 600 US veterans’ electronic medical reports were used in their work, 451 for training and 159 for testing.

Zhao et al. [22] trained a Logistic Regression with LASSO with 100 random split iterations on 550 patients, in which 127 (23%) were manually determined to have axSpA meeting classification criteria and 423 did not. They exploited the Surrogate Assisted Feature Extraction (SAFE) method to extract a list of potential axSpa-related concepts from online resources such as MEDLINE. The SAFE method retrieved four disease concepts, ankylosing spondylitis (AS), sacroiliitis, HLA-B27, and spondylitis. For each patient, the numbers of positive mentions of each axSpA concept were combined with coded data: the number of occurrences of ICD code for AS and the healthcare utilization (i.e., the number of medical encounters in each patient’s record). Then, the authors compared three models: Logistic Regression model; LASSO-LR, and the multimodal automated phenotyping (MAP) [44], an unsupervised approach that classifies phenotypes in EHR data. Although their behaviors were similar in terms of AUC (93.0%, 92.9%, 92.7%), sensitivity (70%, 71%, 78%), specificity (95%, 95%, 94%), and *F*_1_-score (75%, 75%, 79%), the MAP algorithm was slightly better than the others. However, all three methods outperformed methods based on related ICD codes counting. To achieve these performances, they extracted 550 notes (among healthcare provider notes, discharge summaries, and radiology reports), randomly split 100 times into training and test sets.

In 2018, Huhdanpaa et al. [28] developed a pipeline of text pre-processing and concept identification at the document level, using a list of keywords and regular expressions to incorporate spelling variations and negations (NegEx algorithm [39]). They evaluated 458 radiology reports from the Lumbar Imaging with Reporting of Epidemiology (LIRE) study [55], with a prevalence of Type 1 Modic changes approximately of 10%, resulting in a sensitivity of 0.70, a specificity of 0.99, a precision of 0.90, an NPV of 0.96, and a *F*_1_-score of 0.79. Results were reported for a 95% CI.

Tan et al. [18] used a similar approach to identify 26 imaging findings from radiology reports, producing dichotomous predictions for each report, where a positive assignment was made if there was at least one sentence with a keyword that was not modified by a negation term. Also, they applied a multimodal Logistic Regression with elastic-net to n-gram features and Regex and NegEx from the rule-based model (among others), resulting in a hybrid model. They fine-tuned the model hyperparameters on the development subsample with 10-fold cross-validation using a Receiver Operating Characteristic (ROC) loss function. Results, estimated at 95% confidence intervals using bootstrap percentiles on the test set based on 500 iterations, were reported for both the models, in terms of (averaged) sensitivity (0.83, 0.94), specificity (0.97, 0.95), and AUC (0.90, 0.98). They also reported performances in detecting the 8 findings commonly found in subjects without LBP and the 6 findings that are likely clinically more important for LBP. In all the cases, the hybrid model outperformed the rule-based one, especially with regards to sensitivity and AUC metrics.

Building on the same principles, Caton et al. [25] implemented a rule-based model to assess the severity degree of SCS and left and right NFS (including bilateral cases). Each text block, parsed from the “Findings” section of radiology reports, individuates a discrete level from T12-L1 through L5-S1. The 6-point severity grading scale includes “Normal,” “Mild,” “Mild to Moderate,” “Moderate,” “Moderate to Severe,” and “Severe.” Assuming that normal anatomy can be presumed by the absence of specific comment by the radiologist, failure cases (no mentions to the conditions) were identified as the “Normal” class. To accomplish the task, the authors iteratively assembled a dictionary of non-standard terms (e.g., “marked” or “minimally”) to facilitate the mapping of non-standard terms to the grading scale. They reported the accuracy of 94.8% of this system on an annotated random set of 100 LMRI reports, meaning in 93 misclassifications out of 1800 level instances. At the individual levels, NLP accuracy ranged from 86.0%at right L5-S1 to 100% in 5/18 level instances (27.8%). The authors used their system to analyze the effects of age and sex in SCS and NFS, and also to compute a composite severity score in [26].

For what concerns the identification of spinal stenosis, another study has employed the NLP method trained on 5000 manually labeled disc levels extracted from radiology reports [35]. Here, *Lewandrowski et al.* marked both the central canal and the neural foramina based on the radiologist’s report. For the former, the following labels were used: “no signs of abnormality,” “disc bulging without compromise of the thecal sac,” “disc bulging compressing thecal sac (central canal stenosis),” and “disc herniation compressing thecal sac (central canal stenosis).” For the latter, instead, the reports were annotated as “no signs of abnormality,” “left foraminal stenosis,tead, the reports were annotated “right foraminal stenosis,” or “bilateral foraminal stenosis.” The NLP tool was then applied on 17800 disc levels with radiology reports to generate labeled training data for the main deep learning method. The pipeline was similar to the DeepSPINE, proposed by Lu et al. a couple of years earlier [56]. However, no performance for the NLP model was reported in the paper.

For what regards annotation tasks, Galbusera et al. [36] fine-tuned the “bert-base-italian-uncased” pre-trained model to identify 12 spine disorders related findings from radiology reports written in Italian, such as the presence of spinal implants or loss of lordosis. For the training (fine-tuning) phase, they manually annotated 4288 reports, while to evaluate the resulting model they annotated 202 reports. For all findings, the model has generally shown high accuracies and specificities, the former ranging from 0.88 to 0.98 and the latter from 0.84 to 0.99. About the sensitivity metric, the model reported a lower performance, namely 0.5 for the “osteoporosis” and 0.63 for the “fractures” findings. The lower sensitivity can be attributed to the unbalanced nature of the dataset: such radiological findings were more frequently absent than present. About the *F*_1_-score, it ranges from 0.63 (osteoporis, again) to 0.95. The author used the NLP model to train (and evaluate) the main DL algorithm, the ResNet-18 convolutional neural network [57], previously pre-trained on the ImageNet database.^4^

Returning to the classification tasks, Dantes et al. [34] used NLP to identify VTE in the post-operative period. They employed the IDEAL-X tool [40], using both the controlled vocabulary mode and the ML model. They found out that the former was able to reach a better performance in terms of sensitivity (97.2%) and specificity (99.3%), calibrated with 468 and evaluated on 2083 radiology reports. Conversely, with the second mode, they reached a sensitivity of 92% and specificity of 99%. Furthermore, the ML required around 50% of reports to be processed before achieving both metrics to be greater than 95%. For both models, results were reported withing the 95% CI.

Identifying surgical and post-surgical complications is indeed a hot topic in this field. *Karhade et al.* proposed a ML-based pipeline to identify ID [31] and VIs [33]. To extract features, they used the TF-IDF version of bag-of-words and an extreme gradient boosting model. They achieved a high performance for both discrimination and calibration metrics. Also, the Brier Score resulted in being lower than the null Brier Score in each case. Furthermore, in [31] and in [33] they compared their model with gold-standard methodologies exploiting CPT (Current Procedural Terminology) and ICD (International Classification of Disease) codes of the intra-operative events. Their model always outperformed these methodologies, also showing a higher standard net benefit at all thresholds. Ehresman et al. [32] used the same model to statistically analyze 1279 patients. Also, the same research unit exploited the same pipeline in the two prediction tasks, to anticipate reoperation due to wound infection after lumbar discectomy [37] and unplanned readmissions after lumbar fusion [38]. In the first case, the model was trained on 4483 patients and evaluated on 1377 patients, while in the second one totality of 708 patients were used, including 141 patients as the test set. In particular, in [37] their model achieved again better performance than CPT/ICD methodologies. Their studies have shown the adaptability of their proposed pipeline to several tasks, to achieve both classification and prediction outcomes, but limited themselves to searching for more performant models.

From this point of view, the study from Miotto et al. [20] is interesting. They compared several kinds of models, belonging either to rule-based and ML-based (both ML and DL) methods, also including unsupervised models. They aimed to classify whether a patient suffered from acute LBP or not. They evaluated five pipelines. In the first one, a rule-based model was proposed, implemented as a keyword search supported by the NegEx algorithm. About other unsupervised models, they exploited a topic modeling framework, using the Latent Dirichlet Allocation (LDA) model, capturing patterns of word co-occurrences within documents; these word distributions define interpretable topics to which every document can be classified as. Topics referring to acute LBP were manually reviewed, then they considered the maximum likelihood among these topics as the probability that a report referred to acute LBP. About ML models, they implemented Logistic Regression with LASSO, employing BoN or engineered features. Finally, they implemented a convolutional neural network for the DL models category. They also compared the various methods with an ICD baseline, considering as acute LBP all the notes associated with the Low back pain ICD-10 code (M54.5). The rule-based method resulted as the worst model, with recall equal to only 0.03, even worst than the ICD-based one. However, it reached the greatest precision, equal to 0.71. Also, the topic modeling-based approach achieved comparable performance to ICD. The best performing model, however, was the network, achieving a precision of 0.65, recall of 0.73, *F*_1_-score of 0.70, and AUROC and AUPRC equal to 0.98 and 0.72, respectively.

## 5. Discussion

An overview of the analyzed works is reported in Table 2. Interestingly, all the papers included in this review were published in the last few years, with the oldest one dated 2017. Among these, classification and pre-operative tasks were the dominant categories to have been investigated. Also, various domains have been investigated by the authors. Identifying axSpA and spinal stenosis (and related findings) were the most present tasks, and they were investigated in 3 studies each, respectively. For the latter, however, one of the studies focused on the NLP part for annotation. For what concerns intra- and post-operative tasks, *Karhade* (and *Ehresman*) [31–33,37,38] and their corresponding coauthors were particularly productive, constituting approximately one third of included studies on the topic. In addition, the review from Grotto et al. [16] also comes from the same research unit. In fact, they faced several problems, from both the classification and prediction categories. Nonetheless, they always employed the same pipeline to the various task. From a medical point of view, this confirms the adaptability to several domains of their approach, but from an NLP point of view, this sounds more like a limitation, having no improvements of the methodologies between consecutive works. In this regard, the study from Miotto et al. [20] looks more captivating, exploring and comparing different kinds of methods. However, *Karhade*’s team [31,33,37,38] was the only one investigating the interpretability of their system. This, of course, was an effect of choosing XGBoost as the classifier model (Section (h)).

Another thing is the distribution models’ types. Five studies implemented rule-based models, while the rest of the papers used ML-based models. In particular, only two among them exploited DL architectures. As also shown by Miotto et al. [20], the performance of rule-based NLP can be limited. The main reasons are to be found among the complexity of the findings, their ambiguity in reports, and feature sets that are not sufficiently rich. Nonetheless, rule-based methods are intrinsically unsupervised, which means that do not require large annotated datasets, as ML-based ones (especially, when working with deep learning architectures), which is an obstacle to their implementation.

Most of the times, medical researchers used NLP to identify large cohorts of patients in order to conduct their research analyses. In other words, they developed systems to collect datasets by including patients with high probability (according to the developed NLP system) of presenting some condition, in order to conduct their analyses on a larger cohort than they would get with traditional data collection. From this point of view, classification and annotation approaches are even more similar. However, in the annotation tasks the NLP system is employed to develop another system able to identify some spine disorders from radiology images. However, the developed models in the analyzed works may be used by physicians and healthcare providers to improve patients’ care. For example, identifying acute LBP before surgery may provide some insights to the physicians, whether to recommend a therapy against another. Similarly, predicting reoperation in the near future may help healthcare providers to allocate resources in a more efficient way. Analyzing patient outcomes and relative changes in costs applying these systems may be a future research trend. Also, most of the works leverage private databases, which is an issue for comparing various works. It is well known that clinical text is usually full of sensitive content, however, a future direction may be to publicly provide data with respect to the privacy policies. This would help the research community in comparing works with each other.

## 6. Conclusions

NLP is a promising technology that is being extensively investigated in the last year in multiple clinical fields, including spine disorders. Although preliminary, studies on the topic have demonstrated to effectively classifying different conditions and events, label documents and predict outcomes. However, additional studies on larger datasets are needed to better define the role of NLP in the care of patients with spinal disorders.

## Data Availability

The original contributions presented in the study are included in the article/**Supplementary Material**, further inquiries can be directed to the corresponding authors.

## Footnotes

^1^ https://www.project-redcap.org/

^2^ https://lexsrv3.nlm.nih.gov/LexSysGroup/Projects/vtt/current/web/index.html

^3^ https://riverbankcomputing.com/software/pyqt/intro

^4^ http://www.image-net.org/

## References

[1] Burton AK Balagué F Cardon G Eriksen H Henrotin Y Lahad A European guidelines for prevention in low back pain: November 2004. Eur Spine J (2006) 15:s136. 10.1007/s00586-006-1070-3 16550446 PMC3454541

[2] Wu A March L Zheng X Huang J Wang X Zhao J Global low back pain prevalence and years lived with disability from 1990 to 2017: estimates from the global burden of disease study 2017. Ann Transl Med (2020) 8(6):299. 10.21037/atm.2020.02.175 PMC7186678 32355743

[3] Jeffries LJ Milanese SF Grimmer-Somers KA. Epidemiology of adolescent spinal pain: a systematic overview of the research literature. Spine (2007) 32:2630–7. 10.1097/BRS.0b013e318158d70b 17978666

[4] Balagué F Mannion AF Pellisé F Cedraschi C. Non-specific low back pain. Lancet (2012) 379:482–91. 10.1016/S0140-6736(11)60610-7 21982256

[5] Croft P Rigby A Boswell R Schollum J Silman A. The prevalence of chronic widespread pain in the general population. J Rheumatol (1993) 20:710–3. 8496870

[6] Guo H-R Tanaka S Halperin WE Cameron LL. Back pain prevalence in us industry and estimates of lost workdays. Am J Public Health (1999) 89:1029–35. 10.2105/AJPH.89.7.1029 10394311 PMC1508850

[7] Katz JN. Lumbar disc disorders and low-back pain: socioeconomic factors and consequences. JBJS (2006) 88:21–4. 10.2106/JBJS.E.01273 16595438

[8] D’Antoni F Russo F Ambrosio L Vollero L Vadalà G Merone M Artificial intelligence and computer vision in low back pain: a systematic review. Int J Environ Res Public Health (2021) 18:10909. 10.3390/ijerph182010909 34682647 PMC8535895

[9] D’Antoni F Russo F Ambrosio L Bacco L Vollero L Vadalà G. Artificial intelligence and computer aided diagnosis in chronic low back pain: a systematic review. Int J Environ Res Public Health (2022). 10.3390/ijerph19105971 PMC9141006 35627508

[10] Pons E Braun LM Hunink MM Kors JA. Natural language processing in radiology: a systematic review. Radiology (2016) 279:329–43. 10.1148/radiol.16142770 27089187

[11] Yim W-w. Yetisgen M Harris WP Kwan SW. Natural language processing in oncology: a review. JAMA Oncol (2016) 2:797–804. 10.1001/jamaoncol.2016.0213 27124593

[12] Mullenbach J Wiegreffe S Duke J Sun J Eisenstein J Explainable prediction of medical codes from clinical text. [Preprint] (2018). Available at: arXiv:1802.05695.

[13] Bacco L Cimino A Paulon L Merone M Dell’Orletta F. A machine learning approach for sentiment analysis for Italian reviews in healthcare. In: *Computational Linguistics CLIC-IT 2020*. Vol. 630 (2020). p. 16.

[14] Sheikhalishahi S Miotto R Dudley JT Lavelli A Rinaldi F Osmani V. Natural language processing of clinical notes on chronic diseases: systematic review. JMIR Med Inform (2019) 7:e12239. 10.2196/12239 31066697 PMC6528438

[15] Whiting PF Rutjes AW Westwood ME Mallett S Deeks JJ Reitsma JB Quadas-2: a revised tool for the quality assessment of diagnostic accuracy studies. Ann Intern Med (2011) 155:529–36. 10.7326/0003-4819-155-8-201110180-00009 22007046

[16] Groot OQ Ogink PT Oosterhoff JH Beam AL Natural language processing and its role in spine surgery: a narrative review of potentials and challenges. In: *Seminars in spine surgery*. Elsevier (2021). p. 100877.

[17] Brinjikji W Luetmer PH Comstock B Bresnahan BW Chen L Deyo R Systematic literature review of imaging features of spinal degeneration in asymptomatic populations. Am J Neuroradiol (2015) 36:811–6. 10.3174/ajnr.A4173 25430861 PMC4464797

[18] Tan WK Hassanpour S Heagerty PJ Rundell SD Suri P Huhdanpaa HT Comparison of natural language processing rules-based and machine-learning systems to identify lumbar spine imaging findings related to low back pain. Acad Radiol (2018) 25:1422–32. 10.1016/j.acra.2018.03.008 29605561 PMC6162177

[19] Mu J Furlan AD Lam WY Hsu MY Ning Z Lao L Acupuncture for chronic nonspecific low back pain. Cochrane Database Syst Rev. (2020) 12(12):CD013814. 10.1002/14651858.CD013814 PMC8095030 33306198

[20] Miotto R Percha BL Glicksberg BS Lee H-C Cruz L Dudley JT Identifying acute low back pain episodes in primary care practice from clinical notes: observational study. JMIR Med Inform (2020) 8:e16878. 10.2196/16878 32130159 PMC7068466

[21] Robinson PC van der Linden S Khan MA Taylor WJ. Axial spondyloarthritis: concept, construct, classification and implications for therapy. Nat Rev Rheumatol (2021) 17:109–18. 10.1038/s41584-020-00552-4 33361770

[22] Zhao SS Hong C Cai T Xu C Huang J Ermann J Incorporating natural language processing to improve classification of axial spondyloarthritis using electronic health records. Rheumatology (2020) 59:1059–65. 10.1093/rheumatology/kez375 31535693 PMC7850056

[23] Walsh JA Shao Y Leng J He T Teng C-C Redd D Identifying axial spondyloarthritis in electronic medical records of us veterans. Arthritis Care Res (2017) 69:1414–20. 10.1002/acr.23140 27813310

[24] Walsh JA Pei S Penmetsa G Hansen JL Cannon GW Clegg DO Identification of axial spondyloarthritis patients in a large dataset: the development and validation of novel methods. J Rheumatol (2020) 47:42–9. 10.3899/jrheum.181005 30877217

[25] Caton MT Wiggins WF Pomerantz SR Andriole KP. Effects of age and sex on the distribution and symmetry of lumbar spinal and neural foraminal stenosis: a natural language processing analysis of 43,255 lumbar MRI reports. Neuroradiology (2021) 63:959–66. 10.1007/s00234-021-02670-6 33594502 PMC8128837

[26] Caton MT Wiggins WF Pomerantz SR Andriole KP. The composite severity score for lumbar spine MRI: a metric of cumulative degenerative disease predicts time spent on interpretation and reporting. J Digit Imaging (2021) 34(4):811–9. 10.1007/s10278-021-00462-1 PMC8455764 34027590

[27] Jensen TS Karppinen J Sorensen JS Niinimäki J Leboeuf-Yde C. Vertebral endplate signal changes (modic change): a systematic literature review of prevalence and association with non-specific low back pain. Eur Spine J (2008) 17:1407–22. 10.1007/s00586-008-0770-2 18787845 PMC2583186

[28] Huhdanpaa HT Tan WK Rundell SD Suri P Chokshi FH Comstock BA Using natural language processing of free-text radiology reports to identify type 1 modic endplate changes. J Digit Imaging (2018) 31:84–90. 10.1007/s10278-017-0013-3 28808792 PMC5788819

[29] Hassanzadeh H Bell J Bhatia M Puvanesarajah V. Incidental durotomy in lumbar spine surgery; risk factors, complications, and perioperative management. J Am Acad Orthop Surg (JAAOS) (2021) 10:5435. 10.5435/JAAOS-D-20-00210 33539059

[30] Ishikura H Ogihara S Oka H Maruyama T Inanami H Miyoshi K Risk factors for incidental durotomy during posterior open spine surgery for degenerative diseases in adults: a multicenter observational study. PLoS ONE (2017) 12:e0188038. 10.1371/journal.pone.0188038 29190646 PMC5708748

[31] Karhade AV Bongers ME Groot OQ Kazarian ER Cha TD Fogel HA Natural language processing for automated detection of incidental durotomy. Spine J (2020) 20:695–700. 10.1016/j.spinee.2019.12.006 31877390

[32] Ehresman J Pennington Z Karhade AV Huq S Medikonda R Schilling A Incidental durotomy: predictive risk model and external validation of natural language process identification algorithm. J Neurosurg Spine (2020) 33:342–8. 10.3171/2020.2.SPINE20127 32357334

[33] Karhade AV Bongers ME Groot OQ Cha TD Doorly TP Fogel HA Development of machine learning and natural language processing algorithms for preoperative prediction and automated identification of intraoperative vascular injury in anterior lumbar spine surgery. Spine J (2021) 21:1635–42. 10.1016/j.spinee.2020.04.001 32294557

[34] Dantes RB Zheng S Lu JJ Beckman MG Krishnaswamy A Richardson LC Improved identification of venous thromboembolism from electronic medical records using a novel information extraction software platform. Med. Care (2018) 56:e54. 10.1097/MLR.0000000000000831 29087984 PMC5927846

[35] Lewandrowski K-U Muraleedharan N Eddy SA Sobti V Reece BD Ramírez León JF Feasibility of deep learning algorithms for reporting in routine spine magnetic resonance imaging. Int J Spine Surg (2020) 14:S86–97. 10.14444/7131 33298549 PMC7735442

[36] Galbusera F Cina A Bassani T Panico M Sconfienza LM. Automatic diagnosis of spinal disorders on radiographic images: leveraging existing unstructured datasets with natural language processing. Global Spine J (2021). 10.1177/21925682211026910 PMC10416592 34219477

[37] Karhade AV Bongers ME Groot OQ Cha TD Doorly TP Fogel HA Can natural language processing provide accurate, automated reporting of wound infection requiring reoperation after lumbar discectomy? Spine J (2020) 20:1602–9. 10.1016/j.spinee.2020.02.021 32145358

[38] Karhade AV Lavoie-Gagne O Agaronnik N Ghaednia H Collins AK Shin D Natural language processing for prediction of readmission in posterior lumbar fusion patients: which free-text notes have the most utility? Spine J (2021)22(2):272–7. 10.1016/j.spinee.2021.08.002 34407468

[39] Chapman WW Bridewell W Hanbury P Cooper GF Buchanan BG. A simple algorithm for identifying negated findings and diseases in discharge summaries. J Biomed Inform (2001) 34:301–10. 10.1006/jbin.2001.1029 12123149

[40] Zheng S Lu JJ Ghasemzadeh N Hayek SS Quyyumi AA Wang F. Effective information extraction framework for heterogeneous clinical reports using online machine learning and controlled vocabularies. JMIR Med Inform (2017) 5:e12. 10.2196/medinform.7235 28487265 PMC5442348

[41] Devlin J Chang M-W Lee K Toutanova K Bert: Pre-training of deep bidirectional transformers for language understanding. [Preprint] (2018). Available at: arXiv:1810.04805.

[42] Vaswani A Shazeer N Parmar N Uszkoreit J Jones L Gomez AN Attention is all you need. In: *Advances in neural information processing systems* Long Beach, CA, USA (2017). p. 5998–6008.

[43] Blei DM Ng AY Jordan MI. Latent Dirichlet allocation. J Mach Learn Res (2003) 3:993–1022. 10.5555/944919.944937

[44] Liao KP Sun J Cai TA Link N Hong C Huang J High-throughput multimodal automated phenotyping (map) with application to phewas. J Am Med Inform Assoc (2019) 26:1255–62. 10.1093/jamia/ocz066 31613361 PMC6798574

[45] Wilbur WJ Sirotkin K. The automatic identification of stop words. J Inform Sci (1992) 18:45–55. 10.1177/016555159201800106

[46] Lovins JB. Development of a stemming algorithm. Mech Transl Comput Linguistics (1968) 11:22–31.

[47] Harrell Jr FE Lee KL Mark DB. Multivariable prognostic models: issues in developing models, evaluating assumptions and adequacy, and measuring and reducing errors. Stat Med (1996) 15:361–87. 10.1002/(SICI)1097-0258(19960229)15:4<361::AID-SIM168>3.0.CO;2-4 8668867

[48] Stevens RJ Poppe KK. Validation of clinical prediction models: what does the “calibration slope” really measure? J Clin Epidemiol (2020) 118:93–9. 10.1016/j.jclinepi.2019.09.016 31605731

[49] Calster Van McLernon B Van Smeden DJ Wynants M Steyerberg L W. E. Calibration: the achilles heel of predictive analytics. BMC Med (2019) 17:1–7. 10.1186/s12916-018-1207-3 31842878 PMC6912996

[50] Brier GW. Verification of forecasts expressed in terms of probability. Mon Weather Rev (1950) 78:1–3. 10.1175/1520-0493(1950)078<0001:VOFEIT>2.0.CO;2

[51] Vickers AJ van Calster B Steyerberg EW. A simple, step-by-step guide to interpreting decision curve analysis. Diagn Progn Res (2019) 3:1–8. 10.1186/s41512-019-0064-7 31592444 PMC6777022

[52] Harris PA Taylor R Thielke R Payne J Gonzalez N Conde JG A metadata-driven methodology and workflow process for providing translational research informatics support. J Biomed Inform (2009) 42:377–81. 10.1016/j.jbi.2008.08.010 18929686 PMC2700030

[53] Dell’Orletta F Venturi G Cimino A Montemagni S T2k^2^: a system for automatically extracting and organizing knowledge from texts. In: *Proceedings of the Ninth International Conference on Language Resources and Evaluation (LREC’14)* Reykjavik, Iceland (2014). p. 2062–70.

[54] Frantzi KT Ananiadou S. The c-value/nc-value domain-independent method for multi-word term extraction. Journal of Natural Language Processing (1999) 6:145–79. 10.5715/jnlp.6.3_145

[55] Jarvik JG Comstock BA James KT Avins AL Bresnahan BW Deyo RA Lumbar imaging with reporting of epidemiology (lire): protocol for a pragmatic cluster randomized trial. Contemp Clin Trials (2015) 45:157–63. 10.1016/j.cct.2015.10.003 26493088 PMC4674321

[56] Lu J-T Pedemonte S Bizzo B Doyle S Andriole KP Michalski MH Deep spine: automated lumbar vertebral segmentation, disc-level designation, and spinal stenosis grading using deep learning. In: *Machine Learning for Healthcare Conference* (PMLR) (2018). p. 403–19.

[57] He K Zhang X Ren S Sun J Deep residual learning for image recognition. In *Proceedings of the IEEE Conference on Computer Vision and Pattern Recognition* Columbus, OH, USA (2016). p. 770–8.

