# Supplementary material for: Natural language processing in low back pain and spine diseases: A systematic review
Source: Front Surg. 2022 Jul 14;9:957085. doi: 10.3389/fsurg.2022.957085 (PMC9329654; doi:10.3389/fsurg.2022.957085)
Supplement: Supplementary file 1 [file Data_Sheet1_v1.pdf]

frontiers

Natural Language Processing in Low Back Pain and Spine Diseases: A Systematic Review

SUPPLEMENTARY DATA

Study	D1	D2	D3	D4
(Caton et al., 2021a)	Low	Low	Low	High
(Caton et al., 2021b)	High	Low	Low	High
(Miotto et al., 2020)	Low	Low	Low	Unclear
(Walsh et al., 2017)	Unclear	Low	Low	Unclear
(Walsh et al., 2020)	Unclear	Low	Low	Unclear
(Zhao et al., 2020)	Unclear	Low	Low	Unclear
(Huhdanpaa et al., 2018)	Low	Low	Low	Unclear
(Tan et al., 2018)	Low	Low	Low	High
(LewandrowskI et al., 2020)	Unclear	Low	Low	Low
(Galbusera et al., 2021)	Low	Low	Low	Low
(Ehresman et al., 2020)	High	Low	Unclear	Low
(Karhade et al., 2020a)	High	Low	Unclear	Low
(Karhade et al., 2021a)	Low	Low	Unclear	Low
(Dantes et al., 2018)	Low	Low	Unclear	Low
(Karhade et al., 2020b)	Low	Low	Unclear	Low
(Karhade et al., 2021b)	Low	Low	Low	Low

Table S1. Summary of the methodological quality of the included studies regarding the 4 domains (D1, D2, D3, and D4) assessing the risk of bias of the QUADAS-2 score

REFERENCES

Caton MT, Wiggins WF, Pomerantz SR, Andriole KP. Effects of age and sex on the distribution and symmetry of lumbar spinal and neural foraminal stenosis: a natural language processing analysis of 43,255 lumbar mri reports. *Neuroradiology* **63** (2021a) 959–966.

Caton MT, Wiggins WF, Pomerantz SR, Andriole KP. The composite severity score for lumbar spine mri: a metric of cumulative degenerative disease predicts time spent on interpretation and reporting. *Journal of digital imaging* (2021b) 1–9.

Miotto R, Percha BL, Glicksberg BS, Lee HC, Cruz L, Dudley JT, et al. Identifying acute low back pain episodes in primary care practice from clinical notes: Observational study. *JMIR medical informatics* **8** (2020) e16878.

Walsh JA, Shao Y, Leng J, He T, Teng CC, Redd D, et al. Identifying axial spondyloarthritis in electronic medical records of us veterans. *Arthritis care & research* **69** (2017) 1414–1420.

Study	D1	D2	D3
(Caton et al., 2021a)	High	Low	Low
(Caton et al., 2021b)	High	Low	Low
(Miotto et al., 2020)	Low	Low	Low
(Walsh et al., 2017)	Unclear	Low	Low
(Walsh et al., 2020)	Unclear	Low	Low
(Zhao et al., 2020)	Unclear	Low	Low
(Huhdanpaa et al., 2018)	Low	Low	Low
(Tan et al., 2018)	High	Low	Low
(LewandrowskI et al., 2020)	Low	Low	Low
(Galbusera et al., 2021)	Low	Low	Low
(Ehresman et al., 2020)	High	Low	High
(Karhade et al., 2020a)	High	Low	High
(Karhade et al., 2021a)	High	Low	High
(Dantes et al., 2018)	High	Low	High
(Karhade et al., 2020b)	High	Low	High
(Karhade et al., 2021b)	High	Low	High

Table S2. Summary of the methodological quality of the included studies regarding the 3 domains (D1, D2, and D3) assessing the applicability concerns of the QUADAS-2 score

Walsh JA, Pei S, Penmetsa G, Hansen JL, Cannon GW, Clegg DO, et al. Identification of axial spondyloarthritis patients in a large dataset: the development and validation of novel methods. *The Journal of rheumatology* **47** (2020) 42–49.

Zhao SS, Hong C, Cai T, Xu C, Huang J, Ermann J, et al. Incorporating natural language processing to improve classification of axial spondyloarthritis using electronic health records. *Rheumatology* **59** (2020) 1059–1065.

Huhdanpaa HT, Tan WK, Rundell SD, Suri P, Chokshi FH, Comstock BA, et al. Using natural language processing of free-text radiology reports to identify type 1 modic endplate changes. *Journal of digital imaging* **31** (2018) 84–90.

Tan WK, Hassanpour S, Heagerty PJ, Rundell SD, Suri P, Huhdanpaa HT, et al. Comparison of natural language processing rules-based and machine-learning systems to identify lumbar spine imaging findings related to low back pain. *Academic radiology* **25** (2018) 1422–1432.

LewandrowskI KU, Muraleedharan N, Eddy SA, Sobti V, Reece BD, Ramírez León JF, et al. Feasibility of deep learning algorithms for reporting in routine spine magnetic resonance imaging. *International Journal of Spine Surgery* **14** (2020) S86–S97.

Galbusera F, Cina A, Bassani T, Panico M, Sconfienza LM. Automatic diagnosis of spinal disorders on radiographic images: Leveraging existing unstructured datasets with natural language processing. *Global*

Spine Journal (2021) 21925682211026910.

Ehresman J, Pennington Z, Karhade AV, Huq S, Medikonda R, Schilling A, et al. Incidental durotomy: predictive risk model and external validation of natural language process identification algorithm. *Journal of Neurosurgery: Spine* **33** (2020) 342–348.

Karhade AV, Bongers ME, Groot OQ, Kazarian ER, Cha TD, Fogel HA, et al. Natural language processing for automated detection of incidental durotomy. *The Spine Journal* **20** (2020a) 695–700.

Karhade AV, Bongers ME, Groot OQ, Cha TD, Doorly TP, Fogel HA, et al. Development of machine learning and natural language processing algorithms for preoperative prediction and automated identification of intraoperative vascular injury in anterior lumbar spine surgery. *The Spine Journal* **21** (2021a) 1635–1642.

Dantes RB, Zheng S, Lu JJ, Beckman MG, Krishnaswamy A, Richardson LC, et al. Improved identification of venous thromboembolism from electronic medical records using a novel information extraction software platform. *Medical care* **56** (2018) e54.

Karhade AV, Bongers ME, Groot OQ, Cha TD, Doorly TP, Fogel HA, et al. Can natural language processing provide accurate, automated reporting of wound infection requiring reoperation after lumbar discectomy? *The Spine Journal* **20** (2020b) 1602–1609.

Karhade AV, Lavoie-Gagne O, Agaronnik N, Ghaednia H, Collins AK, Shin D, et al. Natural language processing for prediction of readmission in posterior lumbar fusion patients: which free-text notes have the most utility? *The Spine Journal* (2021b).
